# Incidental finding of congenital pericardial defect during vats bullectomy. Tips and tricks to avoid blunder

**DOI:** 10.1016/j.amsu.2021.102806

**Published:** 2021-09-04

**Authors:** Guo Hou Loo, Hafiz Ismail, Muhammad Ishamuddin Ismail, Nur Ayub Bin Md Ali, Mohd Ramzisham Bin Abdul Rahman, Hairulfaizi Haron

**Affiliations:** Department of Surgery, Faculty of MedicineInstitution: Universiti Kebangsaan Malaysia Medical Centre, Jalan Yaacob Latiff, Bandar Tun Razak, 56000, Kuala Lumpur, Malaysia

**Keywords:** Congenital pericardial defect, Asian population, Cardiac surgery, Spontaneous pneumothorax, VATS

## Abstract

The absence of a small portion of the pericardium is termed pericardial defect. This rare entity can be either acquired or congenital. The prevalence of congenital pericardial defect is exceedingly rare, which is approximately 0.002–0.004% of surgical and pathologic series. The most common type is the absence of the entire left side of pericardium, seen in 67% of all patients with a congenital pericardial defect. Other varieties are incredibly uncommon. Congenital pericardial defect has a male preponderance with a male to female ratio of 3:1, and familial occurrence is uncommon.

We report a case of left partial congenital pericardial defect detected incidentally in a 22-year-old man who presented with recurrent left spontaneous pneumothorax. He underwent video-assisted thoracoscopic bullectomy and intraoperatively, we discovered a left partial pericardial defect which exposed the left atrial appendage.

Although generally asymptomatic, patients may present with non-specific cardiac symptoms such as atypical chest pain. Partial pericardial defects have an increased risk of herniation of the whole left atrium, the left atrial appendage or the ventricles. If this occurs, cardiac strangulation may occur, leading to necrosis and sudden death. Cardiac MRI is a sensitive tool and will demonstrate the absence of preaortic pericardial recess.

In conclusion, no surgical intervention is required in cases of congenital pericardial defect, unless the patient is symptomatic due to complications. If detected incidentally during cardiac or thoracic surgery, the best may be to leave it alone.

## Highlights

• A congenital pericardial defect is exceedingly rare, which is approximately 0.002–0.004% of surgical and pathologic series.
• We report a case of partial congenital pericardial defect in a patient who presented with recurrent spontaneous pneumothorax.
• Partial pericardial defects have an increased risk of herniation causing cardiac strangulation.
• Cardiac MRI is a sensitive tool and will demonstrate the absence of preaortic pericardial recess.
• If detected incidentally during an unrelated cardiac or thoracic surgery, the best option may be to leave it alone.

## 1 Introduction

The absence of a small portion of the pericardium is termed pericardial defect. This rare entity can be either acquired or congenital. Acquired pericardial defect often occurs after pericardiotomy to treat an underlying disease such as constrictive pericarditis [1]. The prevalence of congenital pericardial defect is exceedingly rare, which is approximately 0.002–0.004% of surgical and pathologic series [2]. The actual prevalence is likely underestimated as most patients are asymptomatic. However, in thirty to fifty per cent of cases, congenital pericardial defect may be associated with other congenital cardiac abnormalities such as atrial septal defect, persistent ductus arteriosus, bicuspid aortic valve, mitral valve stenosis and tetralogy of Fallot. Pulmonary sequestration and bronchogenic cyst are other associated extracardiac defects [[3], [4], [5]]. The most common type is the absence of the entire left side of pericardium, seen in 67% of all patients with congenital pericardial defect. Other varieties are incredibly uncommon. Congenital pericardial defect has a male preponderance with a male to female ratio of 3:1, and familial occurrence is uncommon [5]. We report a case of left partial congenital pericardial defect detected incidentally in a 22-year-old man who presented with recurrent left spontaneous pneumothorax. This case has been reported in line with the SCARE criteria [6].

## 2 Case presentation

**Fig. 1:**
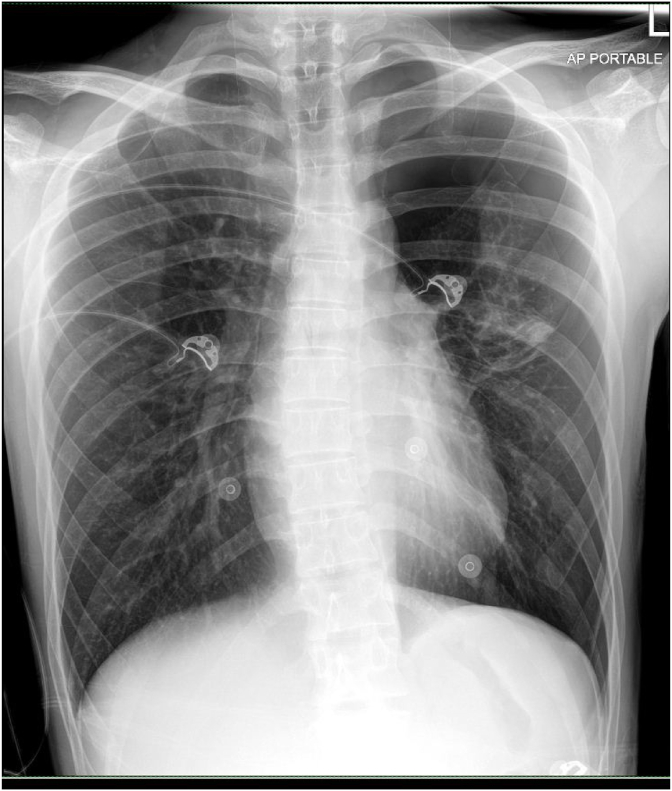
Plain chest radiograph erect showing left sided pneumothorax.

**Fig. 2:**
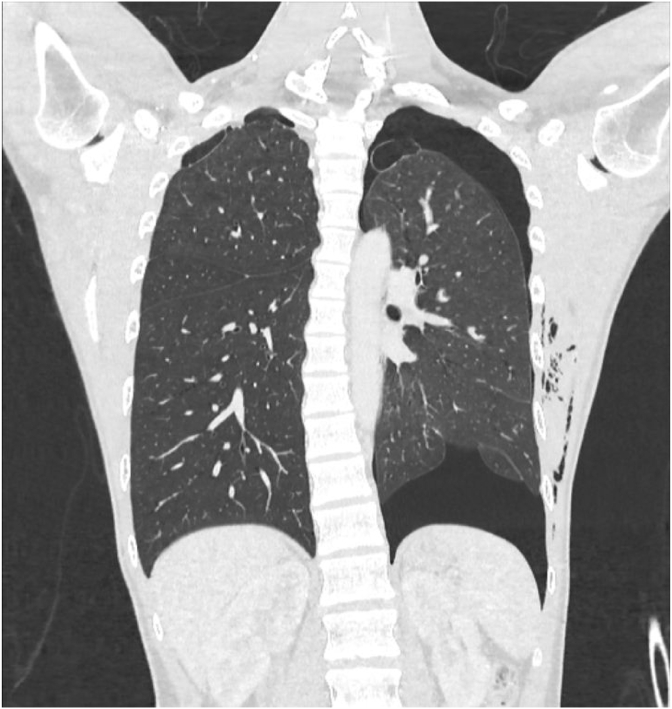
CT Thorax coronal view displaying left pneumothorax with subpleural bullae over apical region of bilateral upper lobes.

**Fig. 3:**
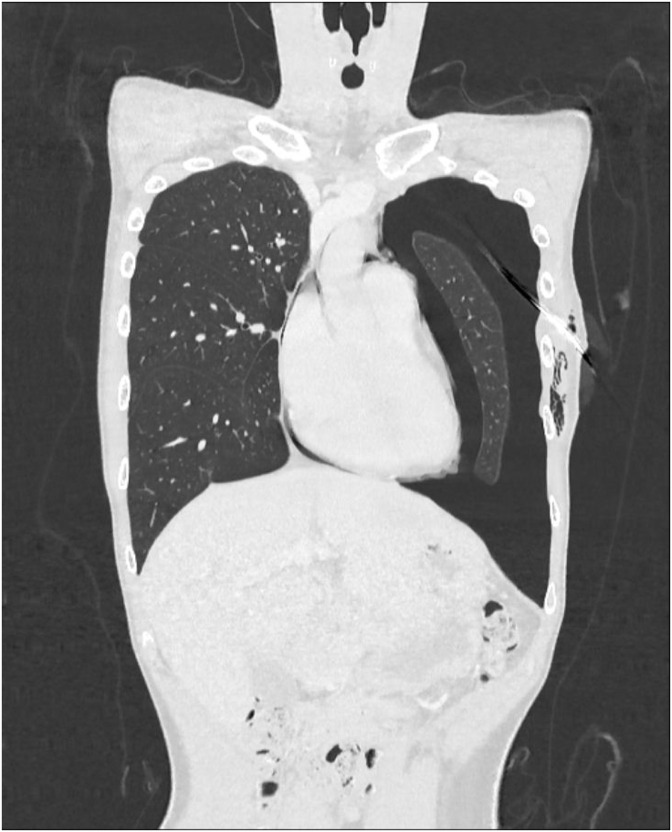
CT Thorax coronal view showing pneumomediastinum over the right heart border.

A healthy 22-year-old man who is an active smoker presented with left-sided chest pain with shortness of breath for a day. Upon clinical examination, he appears mildly tachypneic, and on auscultation, there was reduced air entry over the left thoracic cavity. A plain chest radiograph showed left pneumothorax and a chest tube was inserted[Fig. 1]. Despite chest tube placement, his left pneumothorax was persistent. A CT thorax was performed, and it showed left pneumothorax with a few subpleural bullae at the apicoposterior segment of the left upper lobe. A few subpleural bullae are also present over the apicoposterior segment of the right upper lobe however there was no right pneumothorax[Fig. 2]. Interestingly, there was a presence of pneumomediastinum outlining the great vessels of the heart [Fig. 3]. An echocardiogram was performed, but it was unremarkable, with good left ventricular function, normal chambers and valves.

**Fig. 4:**
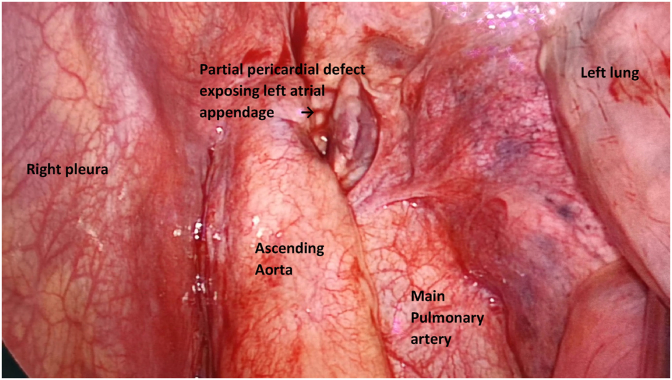
Intraoperative image showing the left partial pericardial defect with left atrial appendage exposed. (as shown by black arrow).

After a multidisciplinary team discussion, he was brought to the theatre for a video-assisted thoracoscopic bullectomy. Intraoperatively, we discovered the cause of the pneumomediastinum. There was a left partial pericardial defect which exposed the left atrial appendage [Fig. 4]. No herniation of the left atrial appendage was seen, and the defect measures 4cm in diameter. Given the large diameter defect with the defect being located superiorly, we decided to leave it alone. The subpleural bullae were removed with a stapling device in the usual manner, and only normal saline was used for irrigation before closure. We were careful to drain all the fluids within the pericardial sac via the positioning of the patient head down and right side up. The patient was nursed in intensive care postoperatively and was extubated after 24hours. He was discharged well and received smoking cessation clinic follow up. During the last clinic follow up twelve months after surgery, he remains well and symptom-free.

## 3 Discussion

Congenital pericardial defect is a rare entity that is usually detected incidentally during cardiac surgery or at autopsy. This defect occurs when the pleuropericardial membrane fails to fuse completely, which may be due to premature atrophy of the left common cardiac vein [5]. The most common type is the absence of the entire left side of pericardium, seen in 67% of all patients with congenital pericardial defect. Partial left defect, complete absence and absent right pericardium varieties are very rare [1]. Congenital pericardial defect has also been associated with Marfan's syndrome, VATER syndrome and Pallister-Killian syndrome [7].

Although generally asymptomatic, patients may present with non-specific cardiac symptoms such as atypical chest pain. This is thought to be secondary to pleuropericardial adhesions and the lack of pericardial cushioning causing undue strain on the great vessels [1]. In contrast, left-sided defects are due to premature obliteration of the left embryonic duct of Cuvier. This leads to incomplete formation of the parietal pericardium. Partial pericardial defects have an increased risk of herniation of the whole left atrium, the left atrial appendage or the ventricles. If this occurs, cardiac strangulation may occur, leading to necrosis and sudden death [1]. Fortunately, most patients with left-sided partial defects are asymptomatic, as in those with complete defects [5]. Cardiac herniation through the defect may lead to chest pain due to compression of the coronary arteries by the rim of remnant pericardium [1]. In patients with partial defects, an unusual symptom of trepopnea may be present. Trepopnea is the occurrence of dyspnoea when lying on one side, but not the other [5].

On clinical examination, systolic murmurs, apical midsystolic clicks and basal ejection murmurs may be present. Electrocardiogram (ECG) typically reveals bradycardia and right bundle branch block. In addition, poor R wave progression and prominent P waves may be present [7]. In our case, there were no ECG abnormalities detected. It is likely because the defect was small and superiorly located, thereby not causing any strain on the heart or herniation.

Cardiac CT and MRI have been used to diagnose this rare entity. Cardiac CT will be able to demonstrate the absence of pericardial tissue and fat but is highly susceptible to motion artefact. Cardiac MRI is a more sensitive tool, and the absence of preaortic pericardial recess is highly suggestive of congenital pericardial defect. However, it has a false positive rate of approximately 10%, especially in patients with less pericardial fat [5]. It is interesting to note that as our patient presented with pneumothorax secondary to ruptured subpleural bullae, the presence of pneumomediastinum is a sign that there is a communication between the pleural and the pericardial cavity.

Fortunately, most cases are asymptomatic, as in our patient. In general, no surgical intervention is required in cases of congenital pericardial defect, unless the patient is symptomatic due to complications. Pericardioplasty has been suggested in patients with non-specific chest pain due to the strain of the great vessels. In cases where cardiac herniation has occurred, pericardiectomy and excision of the herniated left atrial appendage may be required [5]. In our patient, as the defect was an incidental finding, we elect to leave it alone. We were also mindful of the potential hazard that may occur if chemical pleurodesis was performed after bullectomy; hence we chose to perform mechanical pleurodesis instead.

## 4 Conclusion

Congenital pericardial defect is a rare entity with a prevalence of approximately 0.002–0.004% of surgical and pathologic series. In some cases, congenital pericardial defect may be associated with other congenital cardiac and extracardiac abnormalities. Most cases are asymptomatic and do not require any surgical intervention. Cardiac MRI is a sensitive investigative tool for symptomatic patients. If detected incidentally during cardiac or thoracic surgery, the best may be to leave it alone.

## Ethical approval

Ethical approval has been exempted by our institution's ethics committee (The National University of Malaysia's Ethics Committee) as this publication is a case report, provided that patients/patient's next-of-kin have given their informed written consent for the publication of this case report.

## Sources of funding

No source of funding.

## Author statement

Study concepts: Loo Guo Hou, Hafiz Ismail.

Study design: Data acquisition: Loo Guo Hou, Hafiz Ismail.

Quality control of data and algorithms: Loo Guo Hou, Hafiz Ismail.

Data analysis and interpretation: Loo Guo Hou, Hafiz Ismail.

Statistical analysis: Not applicable.

Manuscript preparation: Loo Guo Hou, Hafiz Ismail.

Manuscript editing: Loo Guo Hou, Hairulfaizi Haron, Muhammad Ishamuddin Ismail, Nur Ayub bin Md Ali, Mohd Ramzisham bin Abdul Rahman.

Manuscript review: Loo Guo Hou, Hairulfaizi Haron, Muhammad Ishamuddin Ismail, Nur Ayub bin Md Ali, Mohd Ramzisham bin Abdul Rahman.

## Trial registry number

Not applicable.

## Guarantor

Loo Guo Hou, Hairulfaizi Haron.

## Consent

Written informed consent was obtained from patient and patient's next of kin for publication of this case report and accompanying images. A copy of the written consents is available for review by the Editor-in-Chief of this journal on request.

## Provenance and peer review

Not commissioned, externally peer-reviewed.

## Declaration of competing interest

No conflict of interests.
